# Drug-resistant *Escherichia coli*, Rural Idaho

**DOI:** 10.3201/eid1110.050140

**Published:** 2005-10

**Authors:** Elizabeth L. Hannah, Frederick J. Angulo, James R. Johnson, Bassam Haddadin, Jacquelyn Williamson, Matthew H. Samore

**Affiliations:** * University of Utah School of Medicine, Salt Lake City, Utah, USA; † Centers for Disease Control and Prevention, Atlanta, Georgia, USA; ‡ Veterans Administration Medical Center, Minneapolis, Minnesota, USA; § University of Minnesota, Minneapolis, Minnesota, USA; ¶ Veterans Administration Salt Lake City Health Care System, Salt Lake City, Utah, USA

**Keywords:** Antimicrobial resistance, Escherichia coli, quinolones, dispatch

## Abstract

Stool carriage of drug-resistant *Escherichia coli* in home-living residents of a rural community was examined. Carriage of nalidixic acid–resistant *E. coli* was associated with recent use of antimicrobial agents in the household. Household clustering of drug-resistant *E. coli* was observed. Most carriers of drug-resistant *E. coli* lacked conventional risk factors.

Acquisition of drug-resistant *Escherichia coli* may be influenced by food, exposure to flora of contacts, and use of antimicrobial agents (*1**–**3*). Few community studies have explored the contribution of these mechanisms to dissemination of drug-resistant *E. coli* in healthy persons (*4**–**6*). We examined epidemiologic factors associated with colonization by drug-resistant *E. coli* in home-living volunteers who were not recruited through healthcare settings (*7**,**8*). Resistance to trimethoprim/sulfamethoxazole (TMP/SMZ), nalidixic acid (NA), and extended-spectrum cephalosporins (ESCs) was examined (*9**,**10*).

## The Study

From March to May 2002, a convenience sample of household volunteers was recruited from 1 rural community in Idaho. Consenting adults and parents of children completed an exposure questionnaire. The questionnaire assessed dietary history and livestock contact during the previous month, and other exposures, including antimicrobial drug use and travel outside the United States during the past 6 months. The study was reviewed and approved by the Western Institutional Review Board (Olympia, WA, USA).

Information on antimicrobial drug prescriptions filled by community pharmacies in the preceding year was obtained (beginning March 2001). Pharmacy-documented antimicrobial drug prescriptions were compared with self-reported use. The definition of antimicrobial drug use was either pharmacy documentation of an antimicrobial drug prescription or self-reported use of a named antimicrobial agent obtained from a plausible nonpharmacy (e.g., free sample from a doctor's office) or out-of-area source, with dates of use. Recent antimicrobial drug use was defined as use <30 days before collection of stool swabs.

Study participants were instructed to use a CultureSwab (Becton Dickinson, Franklin Lakes, NJ, USA) to collect fecal material. All samples were refrigerated and transported to the Idaho State Bureau of Laboratories (state laboratory) in Boise, Idaho. At the state laboratory, samples were streaked across 3 MacConkey agar plates, each containing 1 screening antimicrobial agent (16 mg/L TMP/SMZ, 16 mg/L NA, or 2 mg/L cefotaxime). One phenotypically distinct colony type per plate was further analyzed.

Putative *E. coli* colonies were confirmed by using the Microscan system (Dade Behring Inc., Deerfield, IL, USA). Susceptibility was assessed by MIC using broth microdilution (Microscan) for cefpodoxime, ceftazidime, ceftriaxone, and TMP/SMZ and the Etest (AB-BIODISK, Solna, Sweden) for NA. Manufacturer-specified procedures and reference strains were used, along with Clinical and Laboratory Standards (CSLI) (formerly National Committee for Clinical Laboratory Standards [NCCLS]) guidelines. The CSLI/NCCLS criteria were used to classify isolates as resistant to TMP/SMZ, NA, or ESC. Resistance to ESC was defined as resistance to ceftriaxone (MIC >64 μg/mL), ceftazidime (MIC >32 μg/mL), or cefpodoxime (MIC >8 μg/mL) (*11*). A sample was resistant if at least 1 *E. coli* isolate from that sample exhibited the corresponding resistance phenotype.

The primary endpoints were intestinal carriage of *E. coli* resistant to the 3 targeted antimicrobial drug classes. Carriage of NA-resistant and TMP/SMZ-resistant *E. coli* were examined separately by comparing carriers and noncarriers of NA-resistant and TMP/SMZ-resistant *E. coli*. Regression models were constructed in which study participants were divided into 3 mutually exclusive groups: carriers of NA-resistant *E. coli* (either TMP/SMZ resistant or susceptible), carriers of TMP/SMZ-resistant/NA-susceptible *E. coli*, and persons who did not carry either resistance (reference group). Crude and adjusted odds ratios were estimated by using generalized estimating equations to account for household-level clustering. Statistical significance was defined as a p value <0.05. Analyses were performed with Stata version 8.0 (Stata Corporation, College Station, TX, USA).

Stool swabs were received from 517 study participants representing 167 households (Table 1). The prevalence of intestinal carriage of *E. coli* resistant to NA was 3%, to TMP/SMZ 11%, and to ESCs 1%. All 6 ESC-resistant isolates were found so based on their resistance to cefpodoxime. The ceftazidime MIC was in the susceptible range for 5 of these isolates (<4 μg/mL for 2 and 8 μg/mL for 3) and intermediate for 1 isolate (16 μg/mL). The isolate with intermediate susceptibility to ceftazidime also showed intermediate resistance to ceftriaxone (32 μg/mL).

**Table 1:** Characteristics of 517 study participants collected from questionnaires and pharmacy data

Characteristic	No. (%)
Demographic variables	
Male	259 (50)
Income <$35,000/y	339 (66)
Race/ethnicity (n = 497)	
White, non-Hispanic	442 (89)
Hispanic/Latino	30 (6)
American Indian/Alaska Native	9 (2)
Asian	6 (1)
African American	5 (1)
North Hawaiian/Pacific Islander	5 (1)
Age, y	
<6	163 (32)
7–21	103 (20)
22–50	192 (37)
>50	59 (11)
Travel/daycare factors	
Child in daycare/preschool	88 (17)
Travel out of United States in past year	22 (4)
Dietary factors	
Did not eat hamburger in past month	28 (6)
Ate <1 time/wk	48 (9)
Ate 1–2 times/wk	252 (50)
Ate > 2 times/wk	181 (36)
Did not eat chicken in past month	22 (4)
Ate <1 time/wk	59 (28)
Ate 1–2 times/wk	286 (56)
Ate >2 times/wk	147 (29)
Animal exposure	
Live on farm with livestock	5 (1)
Livestock in past month	21 (4)
Cattle	21 (4)
Horses	20 (4)
Sheep	4 (1)
Swine	3 (1)
Poultry	3 (1)
Goats	1 (0)
Household size	
<3 (referent)	76 (46)
3–4	53 (32)
>4	38 (23)

Use of antimicrobial agents was associated with carriage of NA-resistant but not TMP/SMZ-resistant *E. coli*; 6 (16%) of 37 study participants who used antimicrobial agents within 30 days of culture carried NA-resistant *E. coli*, compared with 10 (2%) of 480 participants who did not use antimicrobial agents. However, significance was lost after accounting for household clustering (p = 0.13). Carriage of TMP/SMX-resistant *E. coli* was similar in persons with and without recent use of antimicrobial agents; 5 (14%) of 37 study participants with recent use carried TMP/SMZ-resistant *E. coli* compared with 50 (10%) of 480 persons without recent use (p = 0.84).

A similar pattern was seen for recent use of antimicrobial agents in the household. Overall, 92 (18%) persons resided in a household in which at least 1 member recently used antimicrobial agents. Of these, 11 (12%) of 92 carried NA-resistant *E. coli*, compared with 5 (1%) of 425 in households without recent use. In contrast, the prevalence of carriage of TMP/SMX-resistant *E. coli* was similar in persons with and without recent household use of antimicrobial agents. When we accounted for household clustering, recent use of antimicrobial agents in the household was associated with 9.2-fold increased odds for carriage of NA-resistant *E. coli* (p<0.001). Additionally, the presence of another household member with NA-resistant *E. coli* was associated with 8.8-fold increased odds for NA-resistant *E. coli* carriage (p<0.001), and the presence of another household member with TMP/SMZ-resistant *E. coli* was associated with 2.7-fold increased odds for TMP/SMZ-resistant *E. coli* carriage (p<0.001). Carriage of NA-resistant or TMP/SMZ-resistant *E. coli* was not associated with age, sex, livestock exposure, dietary history, contact with the healthcare system, or travel outside the United States (Table 2). Approximately 94% of persons in the study ate chicken or ground beef in the previous month (Table 1); 14 of 17 persons who did not eat beef or chicken in the previous month were children <5 years of age.

**Table 2:** Comparison of 517 study participants with and without carriage of antimicrobial drug-resistant Escherichia coli, by questionnaire responses and pharmacy data*

Characteristic	Noncarriers (n = 452), no. (%)	TMP/SMZ resistant (n = 49)		NA resistant (n = 16)	
		No. (%)	OR (95% CI)	No. (%)	OR (95% CI)
Demographic variables					
Male	228 (50)	24 (49)	1.0 (0.6–1.8)	9 (56)	1.1 (0.5–2.4)
Income <$35,000/y	69 (13)	10 (20)	1.9 (0.8–4.7)	1 (6)	0.8 (0.1–6.7)
High school education or less	149 (33)	18 (37)	1.3 (0.6–2.9)	3 (19)	0.4 (0.1–2.2)
Hispanic ethnicity	24 (5)	6 (12)	2.6 (0.7– 9.3)	0 (0)	–
Age, y					
<6	144 (32)	16 (33)	Ref	3 (19)	Ref
7–17	71 (16)	5 (10)	1.3 (0.6–2.8)	2 (13)	1.5 (0.7–3.0)
18–50	189 (42)	22 (45)	0.8 (0.4–1.5)	6 (38)	1.3 (0.4–3.7)
>50	48 (11)	6 (12)	0.9 (0.3–2.6)	5 (31)	1.9 (0.3–13.6)
Travel/daycare factors					
Child in daycare/preschool	79 (18)	9 (18)	1.0 (0.5–2.2)	0 (0)	–
Traveled out of United States	17 (4)	1 (2)	0.4 (0.1–1.7)	4 (25)	6.0 (0.9–39.9)
Dietary factors					
Ate hamburger, times/week					
1–2	219 (49)	23 (47)	0.6 (0.3–1.2)	10 (63)	0.7 (0.2–2.0)
>2	164 (37)	14 (29)	0.6 (0.2–1.3)	4 (25)	0.5 (0.2–1.3)
Ate chicken, times/week					
1–2	254 (56)	23 (47)	0.6 (0.3–1.2)	8 (50)	0.8 0.2–2.6
>2	127 (28)	14 (29)	0.6 (0.3–1.5)	7 (44)	1.3 (0.4–4.1)
Household cook	177 (39)	21 (44)	1.0 (0.6–1.7)	7 (44)	0.8 (0.4–1.4)
Primary grocery shopper	169 (37)	20 (41)	1.0 (0.6–1.6)	8 (50)	1.1 (0.6–1.9)
Healthcare/antimicrobial drug use					
Ambulatory visit in past 6 mo	236 (52)	22 (45)	0.8 (0.4–1.3)	9 (56)	1.1 (0.5–2.3)
Diabetic	9 (2)	3 (6)	2.9 (0.7–11.7)	1 (6)	1.5 (0.0–117.4)
Hospitalized in past 6 mo	27 (6)	3 (6)	1.0 (0.3–2.5)	4 (25)	3.4 (0.5–22.4)
Antimicrobial drug use in past 30 d	209 (46)	22 (45)	0.7 (0.2–2.7)	13 (81)	2.6 (0.7–9.7)
Animal exposure in past month					
Livestock	32 (7)	2 (4)	0.5 (0.1–2.1)	1 (6)	1.6 (0.5–4.9)
Cattle	19 (4)	2 (4)	0.8 (0.2–3.4)	0 (0)	–
Horses	18 (4)	1 (2)	0.5 (0.1–4.4)	1 (6)	2.3 (0.7–9.6)
Household size†					
<3	43 (34)	10 (29)	Ref	3 (33)	Ref
3–4	48 (38)	11 (31)	0.7 (0.3–1.6)	3 (33)	1.1 (0.2–5.5)
>4	35 (28)	14 (40)	0.4 (0.2–1.1)	3 (33)	0.4 (0.1–3.4)
Household antimicrobial drug use in past 30 d	21 (17)	5 (14)	0.6 (0.2–1.9)	4 (44)	8.4 (2.4–29.2)

*TMP/SMZ, trimethoprim/sulfamethoxazole; NA, nalidixic acid; OR, odds ratio; CI, confidence interval; Ref, referent. †n = 126 for noncarriers; n = 35 for TMP/SMZ resistant; n = 9 for NA resistant.

The 6 study participants who carried ESC-resistant *E. coli* belonged to 6 separate households. None had used antimicrobial agents within 30 days of culture and only 1 had household use of antimicrobial agents within 30 days. No other epidemiologic or demographic factors distinguished this group. The small number of persons with carriage of ESC-resistant *E. coli* precluded further statistical analysis of this endpoint.

Of the 517 participants, 34% self reported use of an antimicrobial agent during the previous 6 months (Table 3). Of these, 67% had pharmacy documentation of at least 1 antimicrobial agent prescription. However, 22% of the 339 persons who reported not using antimicrobial agents had pharmacy documentation of at least 1 prescription. Of the 178 persons who reported use of >1 antimicrobial agent, 108 (61%) provided the name of the agent. However, the specific drug named matched the drug listed in the pharmacy records for only 29% of the persons. Thirteen persons reported receiving an antimicrobial agent from a nonpharmacy source. Six of the 13 purchased antimicrobial agents in Mexico, 4 received a drug sample from their healthcare provider, and 1 person each received the antimicrobial agent from a dairy, another family member, or a leftover prescription.

**Table 3:** Healthcare/antimicrobial use in 517 study participants collected from questionnaires and pharmacy data*

Characteristic	No. (%)
Ambulatory visit in past 6 mo	266 (52)
Diabetic	13 (3)
Antimicrobial use in past month	37 (7)
No. outpatient visits (past 6 mo)	
0	251 (49)
1	125 (49)
2	59 (23)
3	29 (11)
4	14 (5)
>5	32 (12)
No. hospitalizations (past 6 mo)	
0	483 (93)
1	31 (6)
2	2 (0)
3	1 (0)
No. courses of antimicrobial agents in the past year	
0	333 (64)
1	91 (18)
2	39 (8)
3	19 (4)
4	16 (3)
>5	19 (4)
Antimicrobial classes, no. with >1 course	
TMP/SMZ	
Past month	2 (0.4)
Past year	10 (2)
Fluoroquinolones	
Past month	7 (1)
Past year	17 (3)
Cephalosporins	
Past month	4 (1)
Past year	37 (7)
Penicillin	
Past month	18 (4)
Past year	107 (21)
Macrolide	
Past month	3 (1)
Past year	24 (5)

*TMP/SMZ, trimethoprim/sulfamethoxazole.

## Conclusions

Carriage of *E. coli* resistant to TMP/SMZ was more common than carriage of *E. coli* resistant to NA or ESC. There was striking evidence of household clustering of resistance, consistent with either spread of organisms between persons in close contact or common source acquisition, such as through shared contaminated food (*8**,**12*). Most carriers of drug-resistant *E. coli* did not have exposures previously associated with antimicrobial drug resistance such as travel, contact with the healthcare system, or chronic illness (*13**–**15*).

NA resistance was associated with recent use of antimicrobial agents in the household. Use of antimicrobial agents may have enhanced acquisition of exogenous NA-resistant *E. coli*; alternatively, for persons who had recently taken fluoroquinolones, NA resistance may have emerged during therapy.

Overall, 36% of households had at least 1 member who had received antimicrobial drug treatment within the previous 6 months, illustrating the magnitude of antimicrobial drug selection pressure operating in a community. Self reporting of antimicrobial drug use may be a useful marker of exposure to these drugs when pharmacy records are not available. However, the accuracy with respect to specific drugs was poor.

This study did not convincingly support or refute the hypothesis that contact with contaminated meat contributes to gastrointestinal carriage of drug-resistant *E. coli*. Only a small number of persons reported not eating meat, and those persons lived in households where other members ate meat. Therefore, persons not exposed to meat were not adequately sampled.

The limitations of the study should be acknowledged. Random recruitment of volunteers from the community was not feasible. Since only a single stool specimen was obtained, the duration of carriage of drug-resistant *E. coli* or the timing of its onset in relation to specific exposures could not be determined. The use of thymidine-containing media (MacConkey agar) may have diminished the activity of TMP/SMZ, thereby reducing the sensitivity of the screening for TMP/SMZ resistance.

In conclusion, most home-living residents who carried drug-resistant *E. coli* lacked conventional risk factors. Household-level antimicrobial drug use was associated with carriage of NA-resistant but not TMP/SMZ-resistant *E. coli*. The role of the food supply in promoting dissemination of drug-resistant *E. coli* in human populations warrants more detailed study.
